# Evaluation of Nonlinear Pixel-Wise Squaring for Enhancing Low-Contrast Resolution in CT Imaging

**DOI:** 10.7759/cureus.97479

**Published:** 2025-11-22

**Authors:** Satoru Kawai, Noboru Hojo, Kazuya Abe, Takashi Omino, Shinsuke Kyogoku, Michimasa Suzuki

**Affiliations:** 1 Department of Radiology, Juntendo University Urayasu Hospital, Urayasu, JPN; 2 Department of Radiological Technology, Faculty of Health Science, Juntendo University, Bunkyo, JPN

**Keywords:** acute cerebral infarction, computed tomography, contrast-to-noise ratio, image enhancement, low-contrast detectability, nonlinear image processing, phantom study, squared processing, window setting

## Abstract

Purpose: In hyperacute stroke, early computed tomography (CT) signs are key indicators for diagnosis. However, the earlier the imaging is performed after the onset, the more subtle the blurring of the corticomedullary and insular ribbon boundaries is, making visual identification extremely difficult. This study investigated whether applying nonlinear pixel-wise squaring to CT images could improve low-contrast resolution.

Methods: Using the low-contrast resolution evaluation phantom Catphan 700 (The Phantom Laboratory, Greenwich, NY), four types of images were generated from the acquired CT data: a standard CT image (normal), an image processed with a subtraction of 5 Hounsfield units (HU) from all pixels followed by squaring (Square-5), a squared image without offset (Square), and an image processed with an addition of 5 HU to all pixels followed by squaring (Square+5). Physical evaluations were performed using a task transfer function (TTF), noise power spectrum (NPS), and contrast-to-noise ratio (CNR_LO_). Visual assessment was also conducted.

Results: The subjective visual evaluation revealed that the effectiveness of the non-linear processing was highly dependent on both the scan mode (volume/helical) and the display window setting (wide/narrow).

The proposed Square-5 method demonstrated a significant improvement (p < 0.01) in subjective CNR scores for volume scans, regardless of the window setting. However, it showed no significant improvement for helical scans.

Notably, the Square (no offset) method showed the strongest results when combined with the narrow window setting. Under this specific condition, the Square method significantly improved all three subjective CNR scores (p < 0.05) without a statistically significant negative impact on perceived noise.

Conclusion: The non-linear squaring processing improved low-contrast visibility; however, its effectiveness was highly dependent on both the scan mode and the display window settings. Despite the documented increase in physical noise (NPS), the processing did not cause a corresponding perceived increase in noise. This study demonstrated that under specific conditions, the processing can significantly improve low-contrast visibility, both objectively (CNR_LO_) and subjectively. These optimal conditions were particularly the Square-5 method for volume scans and the offset-free square method combined with a narrow window.

## Introduction

In a study published in The Lancet by the Global Burden of Disease (GBD) 2021 Causes of Death Collaborators in 2024, ischemic stroke was reported to be the second leading cause of death worldwide up to 2019 and the third in 2021 following coronavirus disease [1].

Early treatment after the onset of ischemic stroke can improve patient outcomes; however, delays in initiating treatment often lead to lasting neurological deficits and, in worst cases, death. Therefore, prompt diagnosis is critical. Computed tomography (CT) enables rapid imaging, making it an essential tool in the diagnostic process [2].

In the CT-based diagnosis of ischemic stroke, early CT signs, such as obscuration of the gray-white matter junction, loss of definition of the insular ribbon and lentiform nucleus, effacement of cortical sulci, and the hyperdense middle cerebral artery sign, are key indicators [3]. Furthermore, ASPECTS (Alberta Stroke Program Early CT Score) and PC-ASPECTS (Posterior Circulation ASPECTS) are widely used as methods for assessing the extent of ischemic regions [4,5]. However, detecting subtle signs, such as blurring of the corticomedullary junction or insular cortex, can be extremely difficult. This difficulty is because the difference in CT attenuation between infarcted and normal tissues is often as low as 2-10 Hounsfield units (HU) immediately after onset.

Various methods have been proposed to improve diagnostic accuracy, including narrowing the window width (WW) to enhance contrast [6], using bilateral Z-score comparisons [7], applying model-based iterative reconstruction optimized for the brain [8], and utilizing artificial intelligence-based diagnostic support systems [9,10].

Study objective

The purpose of this study was to evaluate whether applying a nonlinear pixel-wise squaring transformation to CT images can enhance low-contrast visibility. Specifically, we assessed (1) the impact of the transformation on physical image quality metrics, including the task transfer function (TTF), noise power spectrum (NPS), and low-contrast contrast-to-noise ratio (CNR_LO_), and (2) the perceptual effect on image contrast and noise as judged by human observers.

This article was previously posted to the Research Square preprint server on September 17, 2025, and has been assigned a DOI (10.21203/rs.3.rs-7591684/v1).

## Materials and methods

Equipment and imaging conditions

CT was performed using the Aquilion ONE GENESIS Edition (Canon Medical Systems, Otawara, Japan). The scan parameters were adjusted based on our institution’s routine head CT protocol to match the average dose used in clinical practice, with a CT dose index volume of 63 mGy. Both volume and helical scans were performed. The detailed scanning parameters are listed in Table 1.

**Table 1:** Acquisition parameters for volume and helical scans. FOV: field of view; CTDIvol: computed tomography dose index volume.

	Volume scan	Helical scan
Tube voltage	120 kv	
Tube current	320 mA	350 mA
Tube rotation time	1 s	1 s
Detector configuration	0.5 mm x 80 row	
Slice thickness	5 mm	
FOV	24 cm	
Kernel	FC21	
CTDIvol	63 mGy	63.3 mGy

For the phantom studies, we used the Catphan 700 phantom (The Phantom Laboratory, Greenwich, NY), specifically the CTP515 and CTP712 modules. Image analysis was performed using CTmeasure (version 0.97b; Japan Society of CT Technology, Tokyo, Japan) and ImageJ (version 1.53e; National Institutes of Health, Bethesda, MD) [11,12]. Image creation was performed using a custom Python program (version 3.10; Python Software Foundation, Wilmington, DE). In this study, window level (WL) and WW values are expressed in Hounsfield units (HU). For simplicity, the unit is omitted in subsequent descriptions.

Generation of reference and squared images

Reference Image

The pixel-wise squaring used in this study (y = x^2^) is a nonlinear transformation. A key characteristic of this nonlinearity is that the degree of contrast enhancement varies depending on the input signal level, which is determined by the slope of the quadratic function.

This function's slope becomes steeper as the input value moves away from zero. In regions with a steep slope, small differences in the input signal (i.e., contrast) are strongly amplified; however, the noise component present in the image is also significantly amplified. Conversely, in regions with a gentle slope (near zero), signal enhancement is modest, but noise amplification is suppressed.

The low-contrast resolution, as measured by the contrast-to-noise ratio (specifically CNR_LO_), is determined by this trade-off between signal gain and noise amplification. Therefore, maximizing CNR_LO_ requires finding an optimal balance point.

To investigate this trade-off, we introduced an "offset process" (addition or subtraction of HU values) before applying the square transformation. The purpose of this offset was to intentionally shift the CT values of the region of interest (ROI) onto different regions of the quadratic function's slope.

Based on this rationale, four types of images were prepared for analysis: a standard CT image (normal), an image processed by subtracting 5 HU from all pixels followed by squaring (Square-5), a squared image without offset (Square), and an image processed by adding 5 HU to all pixels followed by squaring (Square+5).

The offset value was set to 5 HU to avoid negative pixel values after the subtraction. This was determined based on the average phantom HU value in the CTP515 module (mean = 10 HU; minimum = 5 HU, and maximum = 18 HU).

Acquisition of Reference Images and Squared Image Generation

Normal images were acquired by performing five consecutive scans under the conditions listed in Table 1. The image array was obtained from the DICOM tag of each image, and these arrays were then averaged to create a summation-averaged image. This image was saved as a RAW file and used for analysis.

Two challenges were addressed in the square transformation. First, CT values ≤ 0 become positive after squaring, which can lead to distortion. Second, because CT images are stored as 16-bit data, squaring values greater than 255 can cause overflow. To mitigate these issues, pixel values ≤ 0 were replaced with 0, and values ≥ 255 were capped at 255 before applying the square transformation. After these replacements, squaring was applied, and summation-averaged images were created and saved as RAW files for analysis.

For the Square-5 and Square+5 images, all pixel values from the five acquired scans were first offset by -5 HU or +5 HU, respectively. These offset images then underwent the same replacement and square processing steps described above.

Evaluation of physical characteristics

The evaluation items included the TTF, NPS, low-contrast object-specific contrast-to-noise ratio (CNR_LO_), and visual assessment [13].

TTF

**Figure 1:**
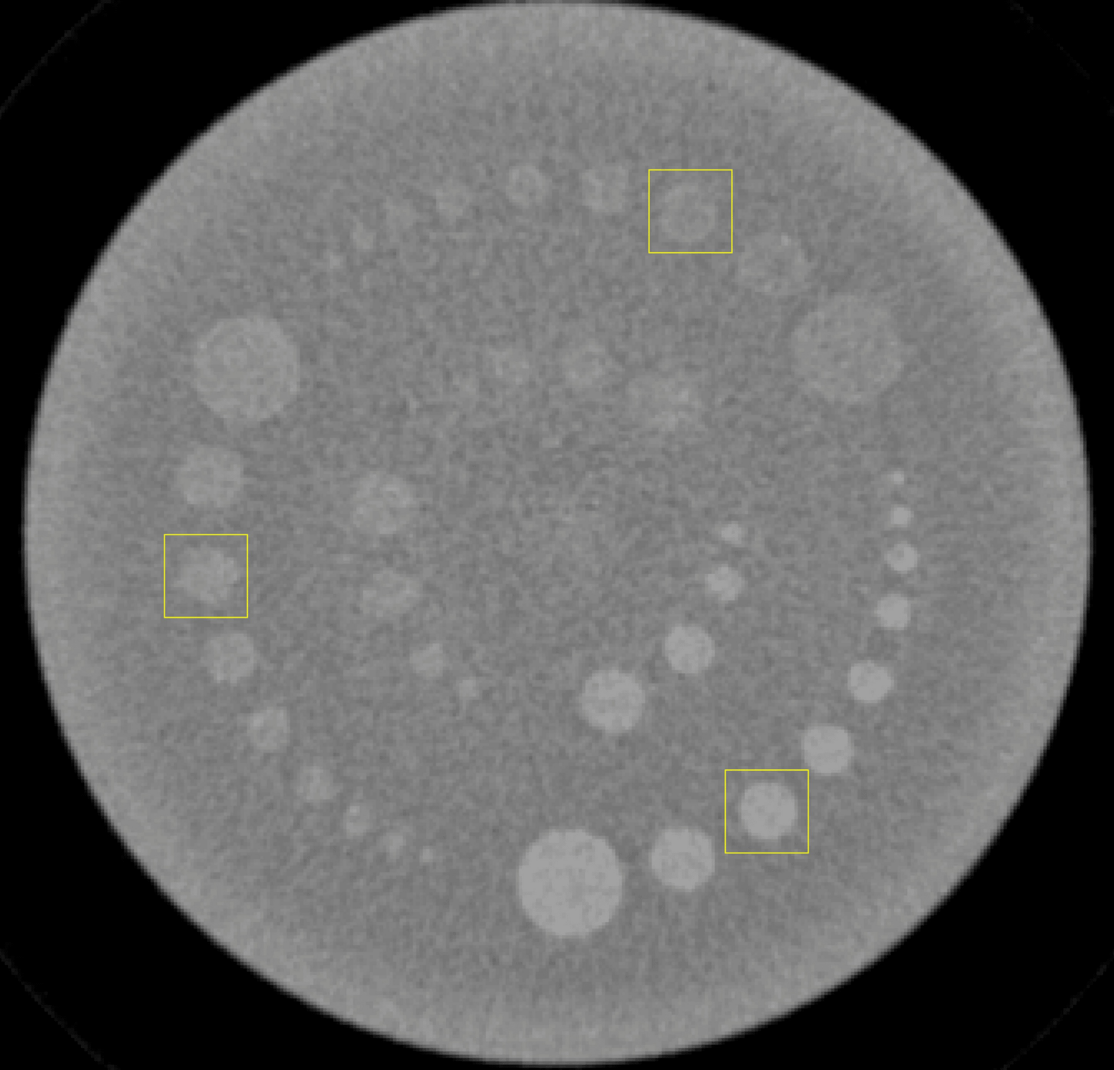
Image of the region of interest used for evaluating the task transfer function.

The TTF was calculated using the circular edge method [14,15]. The analysis was performed on an 8 mm rod diameter in the CTP515 module at nominal target contrast levels (hereafter referred to as contrast levels) of 1.0%, 0.5%, and 0.3%. A 25 × 25-pixel ROI was placed adjacent to the rod at each contrast level. The ROI placement is shown in Figure 1.

NPS

**Figure 2:**
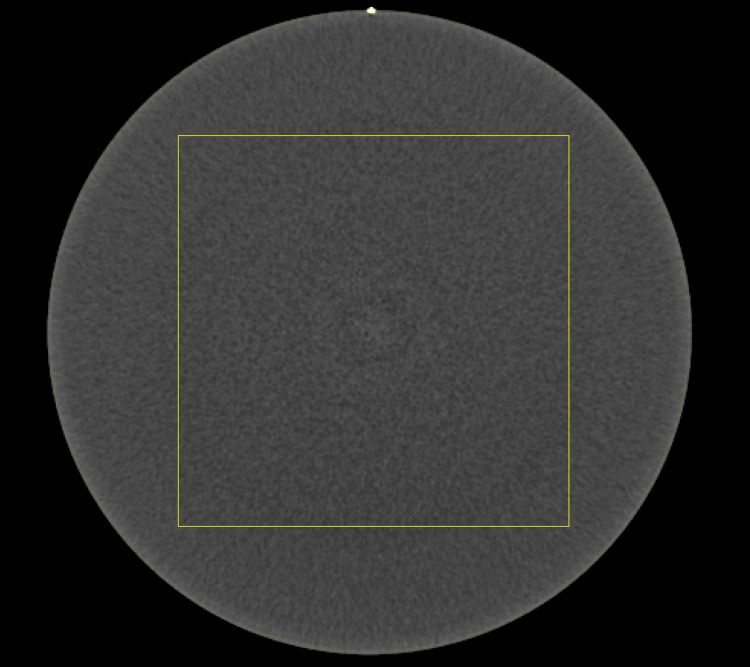
Image of the region of interest used for evaluating the noise power spectrum.

For NPS analysis, the radial frequency method was used [16]. A 256 × 256-pixel ROI was placed at the center of the CTP712 module for measurements (Figure 2).

CTmeasure was used to analyze both the TTF and NPS [11].

CNR_LO_

**Figure 3:**
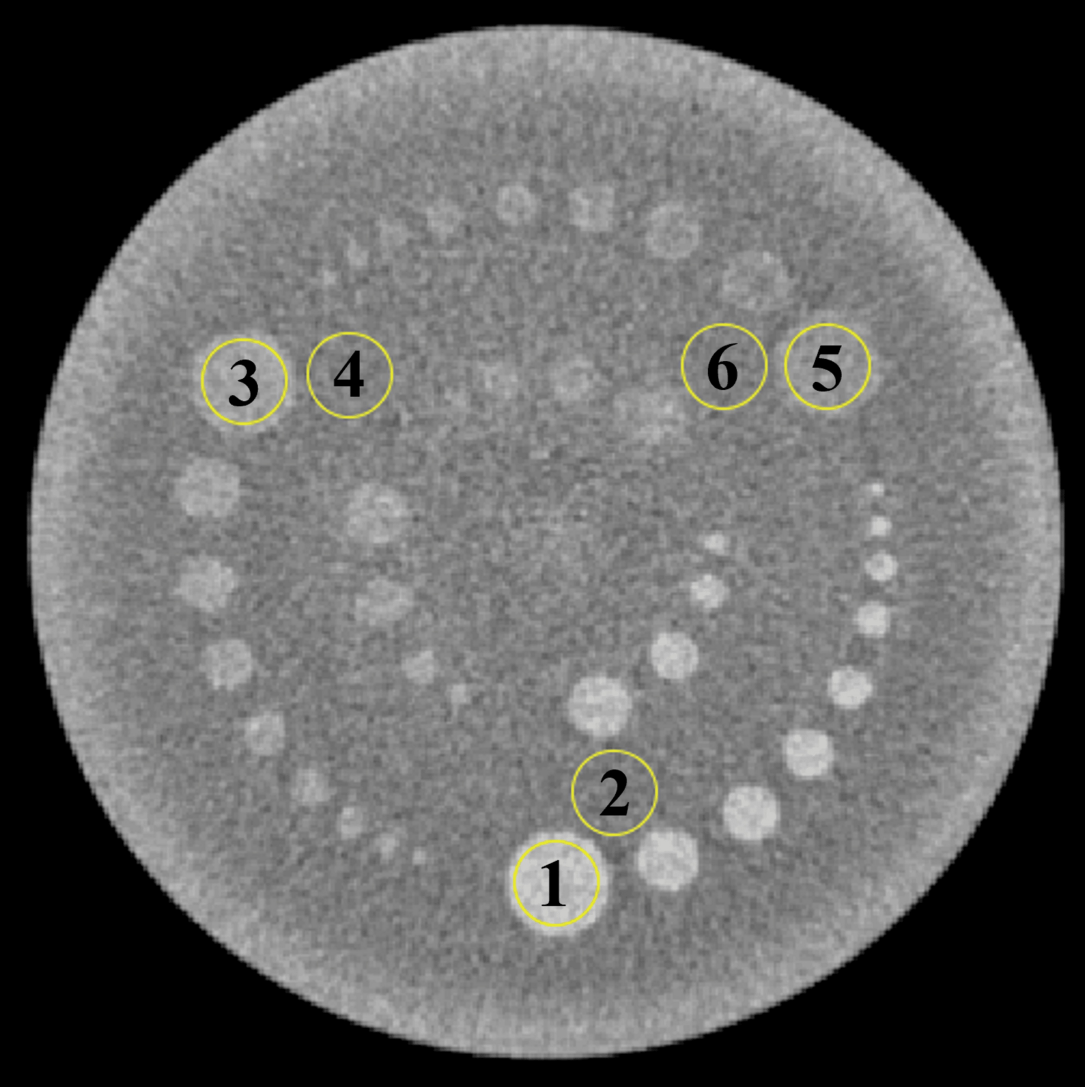
Image of the region of interest used for evaluating the low-contrast object-specific contrast-to-noise ratio.

To measure the CNR_LO_, a circular ROI with a diameter of 12 mm was placed on the 15 mm rod in the CTP515 module. The ROI placement is shown in Figure 3.

An identical ROI was placed in the background region, and the mean values were calculated for contrast levels of 1.0%, 0.5%, and 0.3%. The CNR_LO_ was calculated using the following equation:

$$CNR_{LO}(\bar{u}) = \frac{ROI_{M} - ROI_{B}}{\sqrt{NPS(\bar{u})}} \tag{1}$$

Where ROI_M_ and ROI_B_ represent the mean values of the rod and the background, respectively. The term u represents the spatial frequency corresponding to the pin (task), and $NPS(\bar{u})$ is the value of the noise power spectrum at that specific frequency.

For this measurement, data from the 15 mm (u = 0.03 lp/mm) pin were used. This is because the purpose of this study is to evaluate low-contrast detectability. ROI measurements were performed using the ImageJ software.

Visual Assessment

A visual assessment was conducted using the normal image and the three types of squared images: Square-5, Square, and Square+5. The evaluation items included noise and contrast at nominal target contrast levels of 1.0%, 0.5%, and 0.3%. A paired comparison method was employed for statistical testing, with the significance level set at 0.05.

For image display, WL and WW were set to 50 and 40, respectively, for the normal image. When the squared values of WL and WW are applied directly to the squared images, the pixel value distribution becomes skewed toward higher CT values owing to the nonlinear nature of the squaring process. As a result, using the squared value of WW for the display causes high- and low-CT pixels to fall outside the display range, effectively narrowing the visible window compared to the normal image.

To account for this nonlinearity, we defined WW_Low_ and WW_High_ as 30 and 70, respectively, based on the WW range of the normal image. Their squared values were used to calculate the WW (as the difference) and WL (as the midpoint) for the squared images. For Square-5 and Square+5, the WW_Low_ and WW_High_ values were offset by ±5 HU prior to squaring, and WW and WL were determined using the same approach. The specific WL and WW settings for each image type are summarized in Table 2.

**Table 2:** Settings of WL and WW for squared images. For normal images, the window level (WL) and window width (WW) were set to 50 and 40 Hounsfield units (HU), respectively. For squared images, WW was calculated as the difference between the squared lower (30 HU) and upper (70 HU) limits, and WL as their midpoint. For Square-5 and Square+5, ±5 HU offsets were applied before squaring.

Image type	WW_Low_ (HU)	WW_High_ (HU)	WL (HU)	WW (HU)
Normal	30	70	50	40
Square-5	625 (25^2^)	4,225 (65^2^)	2,425	3,600
Square	900 (30^2^)	4,900 (70^2^)	2,900	4,000
Square+5	1,225 (35^2^)	5,625 (75^2^)	3,425	4,400

In addition, based on the report by Nagashima et al., which demonstrated improved diagnostic performance with narrower window settings, we conducted a visual assessment under narrow window conditions, in which the WW was reduced to 50% of its original value [6].

The visual assessments were performed by 11 qualified radiologic technologists using a paired, side-by-side comparison method.

In each trial, observers were simultaneously presented with the standard normal image (as the reference) and one of the three processed images (Square-5, Square, or Square+5). To minimize bias, the presentation order of the processed images was randomized for each observer.

The observers were asked to evaluate the processed image relative to the reference image based on two criteria: (1) the perceived "Noise" level, and (2) the "Contrast" visibility of the rods (nominal contrast levels of 1.0%, 0.5%, and 0.3%) against the background.

The scoring was performed using a five-point scale as follows: +2 = clearly better, +1 = slightly better, 0 = equivalent, -1 = slightly worse, and -2 = clearly worse.

The statistical significance between the Normal image and each processed image was evaluated using the Wilcoxon signed-rank test.

This non-parametric test was selected because the subjective evaluation data consists of paired ordinal scores (a five-point scale), making it the statistically appropriate method for this type of data.

All statistical analyses, including the Wilcoxon signed-rank test and the generation of the box plots, were performed using SPSS software (version 29.0.0.0, IBM Corp., Armonk, NY).

## Results

TTF

**Figure 4:**
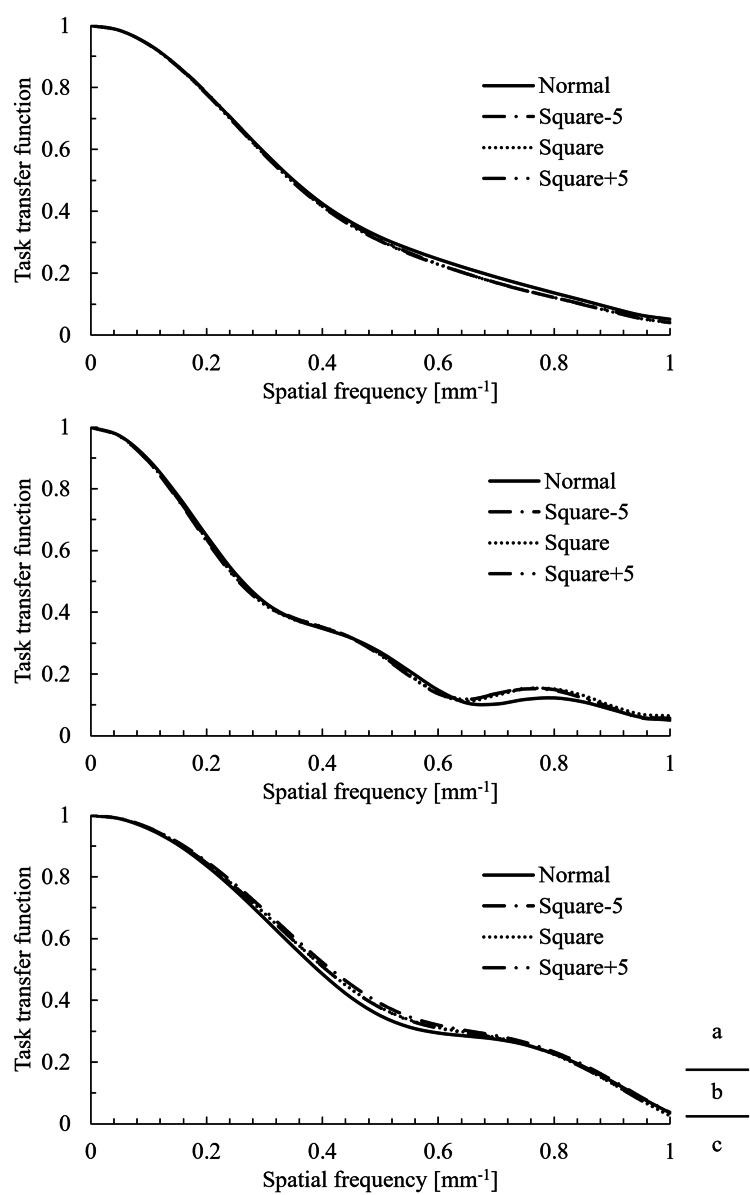
TTF results from the volume scans. Task transfer function (TTF) at nominal target contrast levels of (a) 1.0%, (b) 0.5%, and (c) 0.3%.

**Figure 5:**
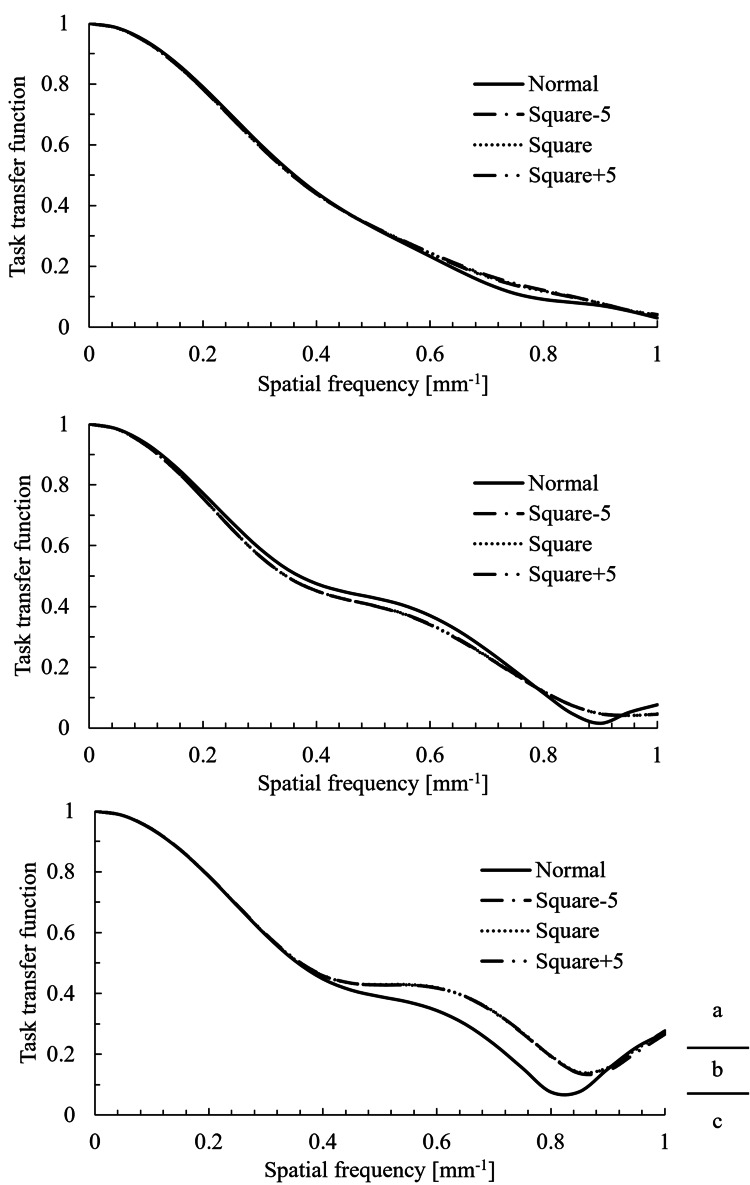
TTF results from the helical scans. Task transfer function (TTF) at nominal target contrast levels of (a) 1.0%, (b) 0.5%, and (c) 0.3%.

The TTF results for the volume and helical scans are shown in Figure 4 and Figure 5, respectively. The 50% and 10% TTF values are summarized in Table 3.

**Table 3:** Comparison of 50% and 10% TTF between volume and helical scans. TTF: task transfer function.

		Volume		Helical	
	Image type	50% TTF	10% TTF	50% TTF	10% TTF
1.00%	Normal	0.35	0.87	0.36	0.78
	Square-5	0.35	0.85	0.36	0.85
	Square	0.35	0.85	0.36	0.85
	Square+5	0.35	0.85	0.36	0.85
0.50%	Normal	0.26	0.87	0.37	0.81
	Square-5	0.25	0.88	0.35	0.82
	Square	0.25	0.89	0.35	0.82
	Square+5	0.26	0.89	0.35	0.82
0.30%	Normal	0.39	0.94	0.36	0.79
	Square-5	0.42	0.94	0.36	-
	Square	0.4	0.93	0.36	-
	Square+5	0.41	0.93	0.36	-

For the helical scan at the 0.3% contrast level, a marked difference was observed between the normal and squared images in the high-frequency range of the TTF curve. In this region, the TTF did not fall below 0.1, making it impossible to determine the 10% TTF. In other regions, no significant differences were observed in TTF shape, 50% TTF, or 10% TTF among the image types.

NPS

**Figure 6:**
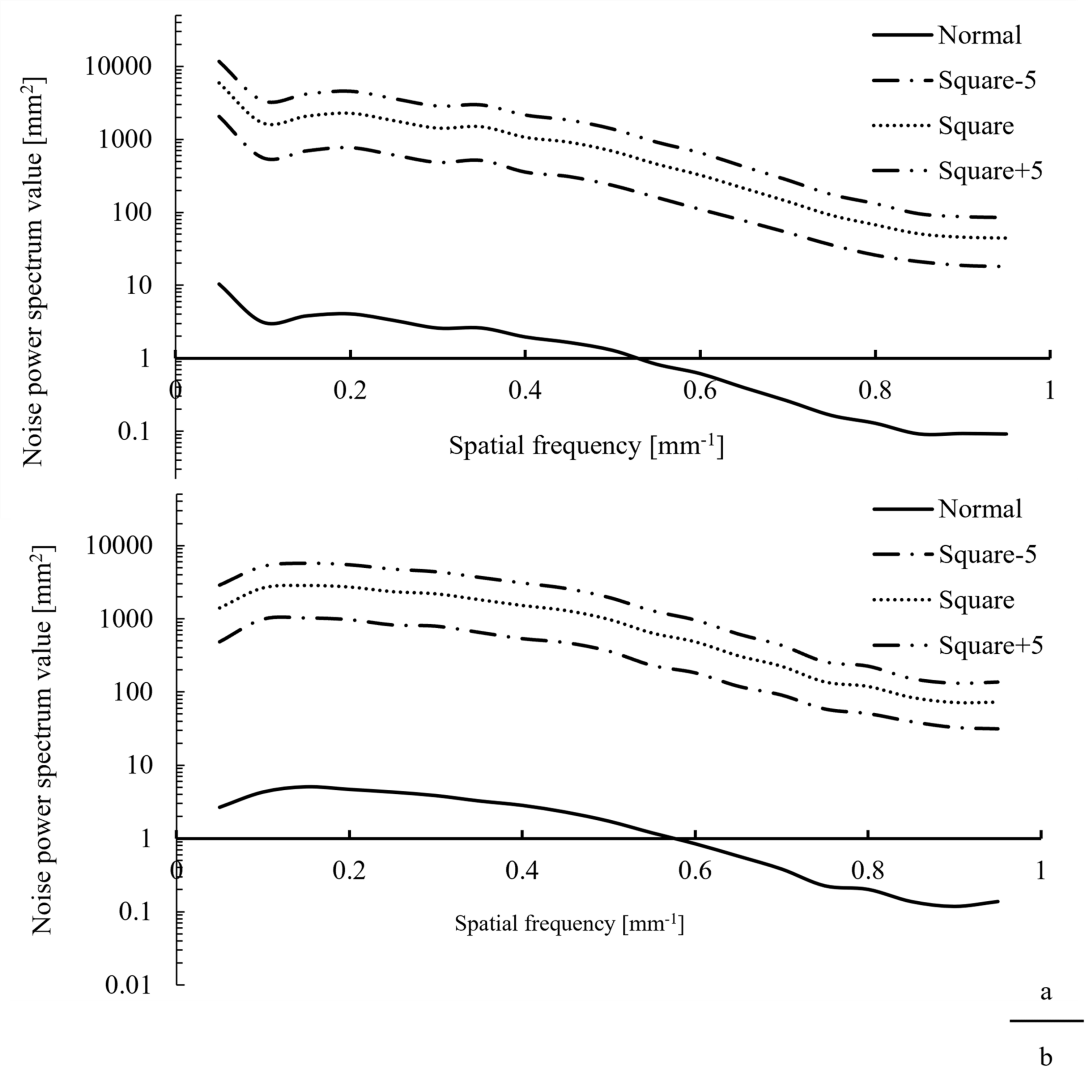
NPS from volume and helical scans. (a) Noise power spectrum (NPS) of volume scan. (b) NPS of helical scan.

The NPS results are shown in Figure 6.

Although there were changes in the absolute values for both the volume and helical scans, no substantial differences were observed in the overall shapes of the NPS curves between the normal and squared images.

For the volume scan, the NPS values at 0.3 lp/mm (corresponding to the 15 mm CTP515 module pin) were 2.6, 485.5, 1417.7, and 2870.9 for the normal, Square-5, Square, and Square+5 images, respectively. Compared to the normal image, these values were 186.7, 545.3, and 1104.2 times higher, respectively.

For the helical scan, the values at 0.3 lp/mm were 3.8, 793.7, 2194.2, and 4354.1 for the normal, Square-5, Square, and Square+5 images, respectively. Compared to the normal image, these values were 208.9, 577.4, and 1145.8 times higher, respectively.

A slightly greater increase in noise was observed in the helical scans than in the volume scan.

CNR_LO_

**Figure 7:**
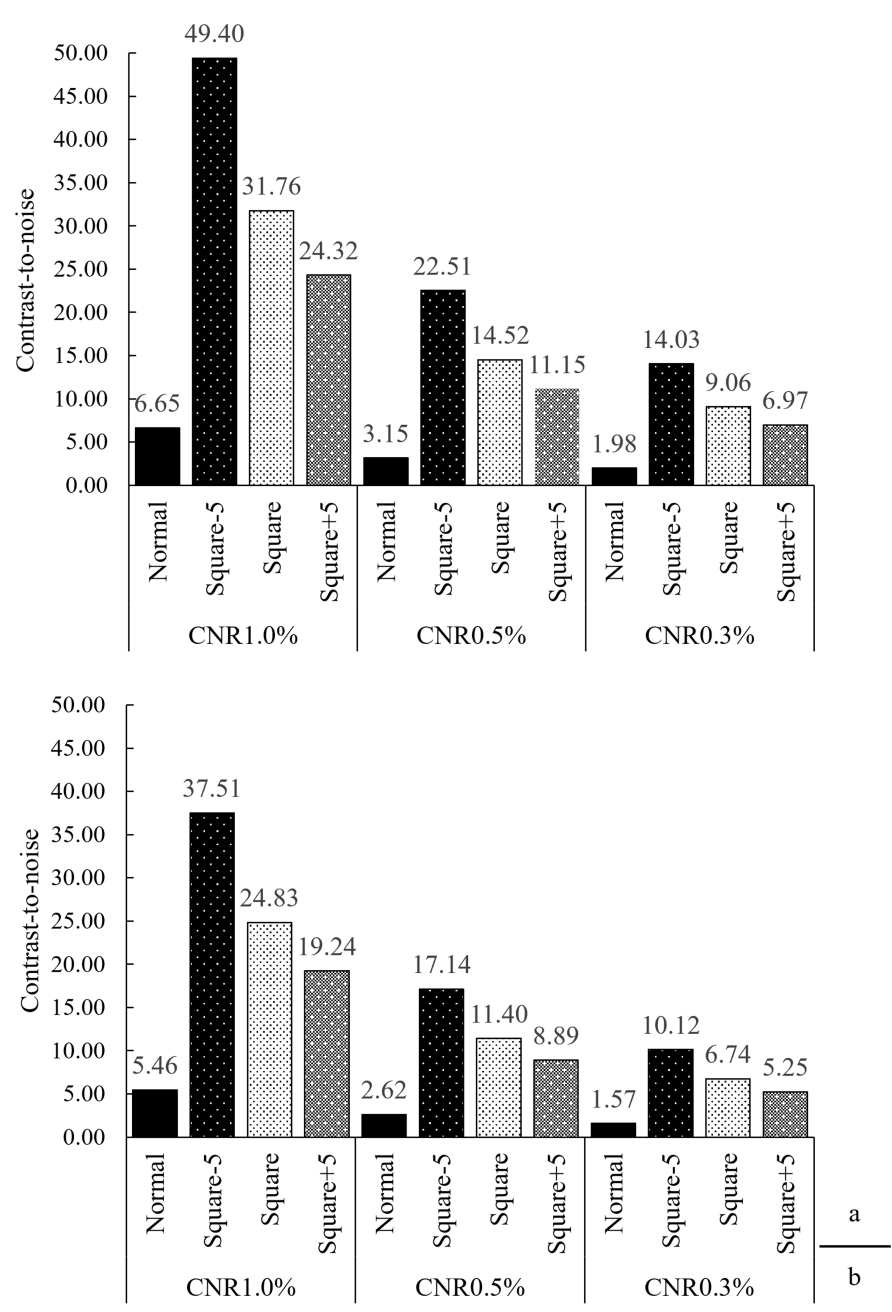
Comparison of low-contrast object-specific CNRLO between original and squared computed tomography images. (a) Contrast-to-noise ratio (CNR_LO_) comparison using volume scan. (b) CNR_LO_ comparison using helical scan.

Table 4 shows the detailed results of the measured phantom values, and Figure 7 presents their graphical representation.

**Table 4:** Mean, SD, and CV of ROIs in phantom images. Mean: mean CT values; SD: standard deviation; CV: coefficient of variation (CV = SD/Mean); ROI: region of interest deviation.

			Volume			Helical		
			Mean	SD	CV (%)	Mean	SD	CV (%)
1.00%	Normal	Pin	61	1.8	3.0	60	1.9	3.2
		Background	50	1.6	3.2	49	1.7	3.5
	Square-5	Pin	3152	200.0	6.3	3034	204.2	6.7
		Background	2063	145.7	7.1	1977	152.3	7.7
	Square	Pin	3737	217.8	5.8	3608	222.7	6.2
		Background	2542	161.8	6.4	2455	169.5	6.9
	Square+5	Pin	4373	235.6	5.4	4232	241.3	5.7
		Background	3070	177.9	5.8	2963	186.7	6.3
0.50%	Normal	Pin	56	1.5	2.7	55	2.0	3.6
		Background	51	1.6	3.1	50	2.1	4.2
	Square-5	Pin	2644	156.1	5.9	2532	202.0	8.0
		Background	2148	142.8	6.6	2049	182.9	8.9
	Square	Pin	3183	171.3	5.4	3059	222.0	7.3
		Background	2636	158.2	6.0	2525	203.2	8.0
	Square+5	Pin	3771	186.5	4.9	3635	241.9	6.7
		Background	3173	173.7	5.5	3050	223.5	7.3
0.30%	Normal	Pin	54	1.5	2.8	53	2.0	3.8
		Background	51	1.5	2.9	50	2.1	4.2
	Square-5	Pin	2448	151.0	6.2	2313	191.3	8.3
		Background	2139	134.5	6.3	2028	185.5	9.1
	Square	Pin	2966	166.3	5.6	2817	211.1	7.5
		Background	2625	149.1	5.7	2501	206.0	8.2
	Square+5	Pin	3535	181.7	5.1	3371	230.9	6.8
		Background	3162	163.7	5.2	3025	226.5	7.5

For both volume and helical scans, CNR_LO_ values increased in the order of Square-5, Square, and Square+5 across all contrast levels, showing significantly higher values than those for the normal image. This objective improvement in CNR_LO_ aligned with the results of the subjective visual assessment (shown later), which also demonstrated a significant enhancement in perceived low-contrast visibility.

Visual assessment

**Figure 8:**
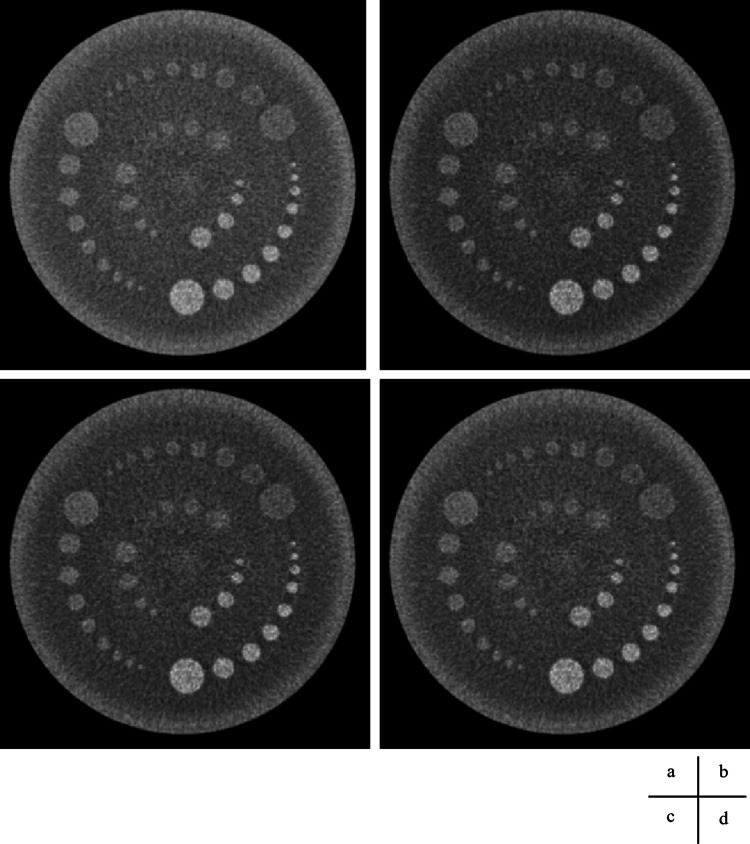
Volume scan and processed images using square transformations, displayed with a wide window. (a) Original CT image: window level (WL) = 50 Hounsfield units (HU); window width (WW) = 40 HU. (b) Square-5 image: WL = 2425 HU; WW = 3600 HU. (c) Square image: WL = 2900 HU; WW = 4000 HU. (d) Square+5 image: WL = 3425 HU; WW = 4400 HU.

**Figure 9:**
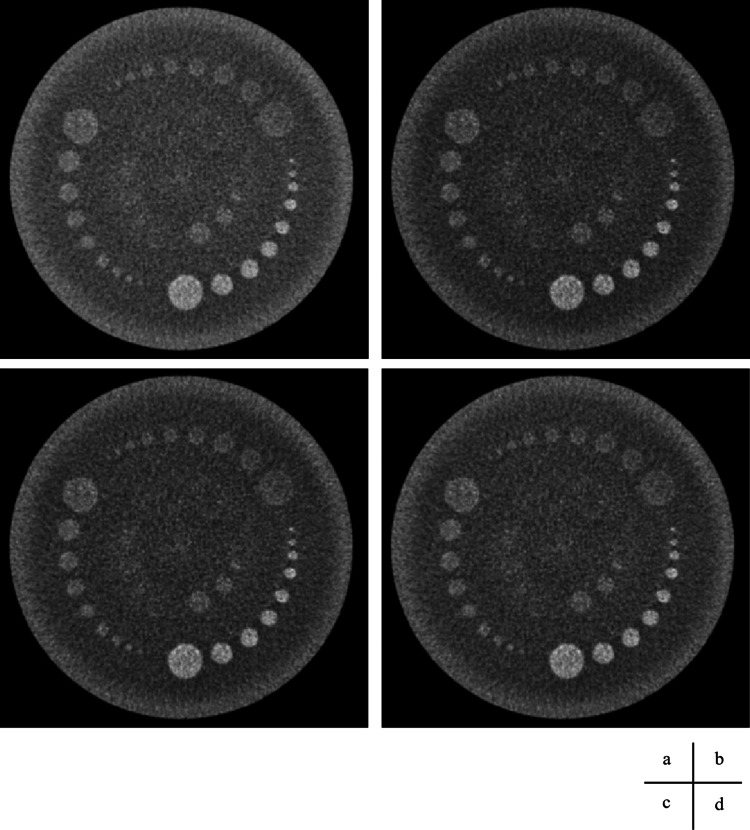
Helical scan and processed images using square transformations, displayed with a wide window. (a) Original CT image: window level (WL) = 50 Hounsfield units (HU); window width (WW) = 40 HU. (b) Square−5 image: WL = 2425 HU; WW = 3600 HU. (c) Square image: WL = 2900 HU; WW = 4000 HU. (d) Square+5 image: WL = 3425 HU; WW = 4400 HU.

**Figure 10:**
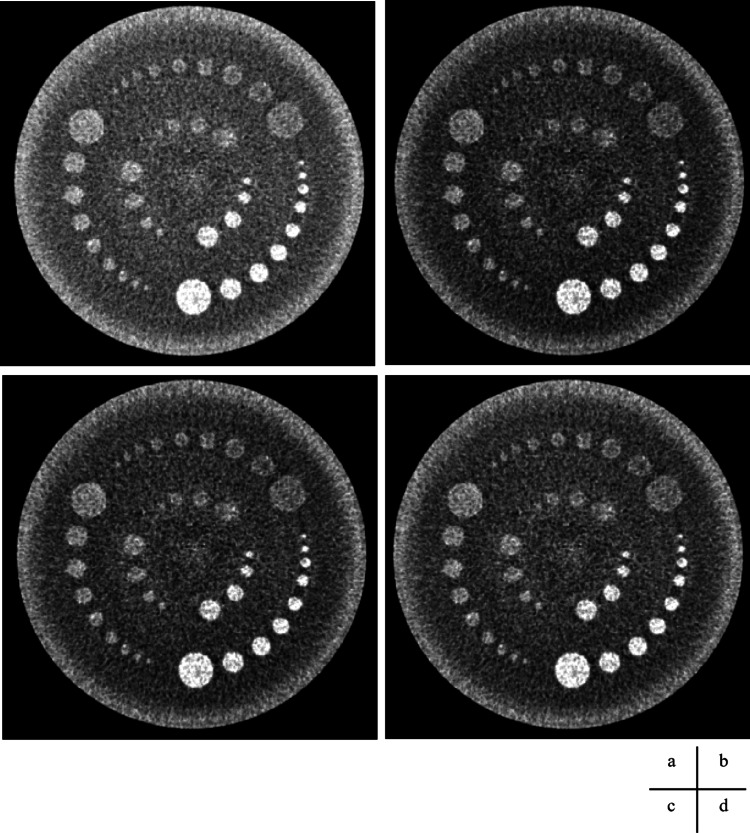
Volume scan and processed images using square transformations, displayed with a narrower window width to enhance contrast. (a) Original image: window level (WL) = 50 Hounsfield units (HU); window width (WW) = 20 HU. (b) Square−5 image: WL = 2425 HU; WW = 1800 HU. (c) Square image: WL = 2900 HU; WW = 2000 HU. (d) Square+5 image: WL = 3425 HU; WW = 2200 HU.

**Figure 11:**
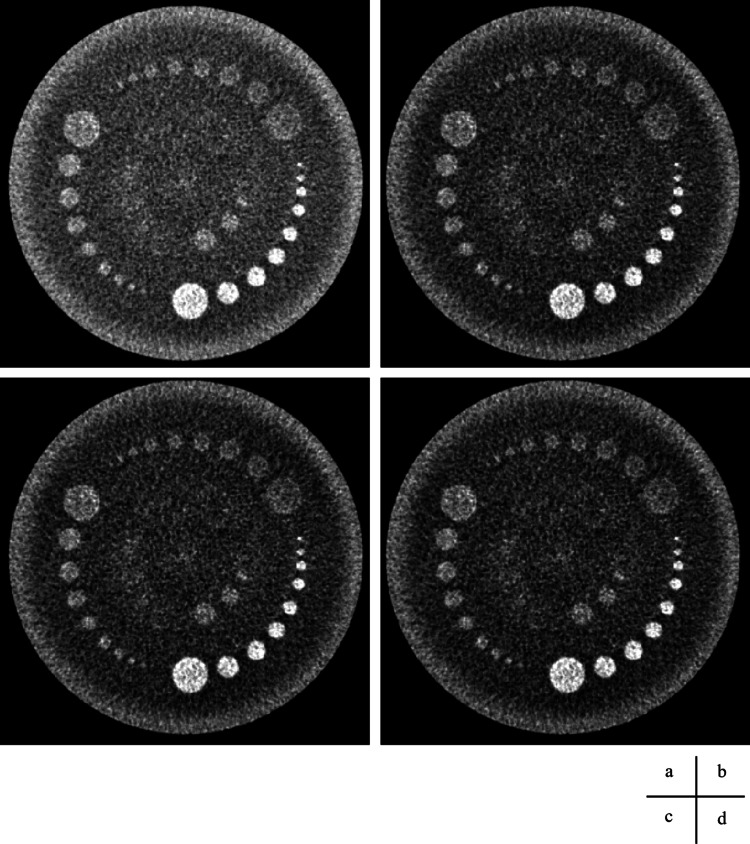
Helical scan. Computed tomography image and processed images using square transformations, displayed with a narrower window width to enhance contrast. (a) Original image: window level (WL) = 50 Hounsfield units (HU); window width (WW) = 20 HU. (b) Square−5 image: WL = 2425 HU; WW = 1800 HU. (c) Square image: WL = 2900 HU; WW = 2000 HU. (d) Square+5 image: WL = 3425 HU; WW = 2200 HU.

**Figure 12:**
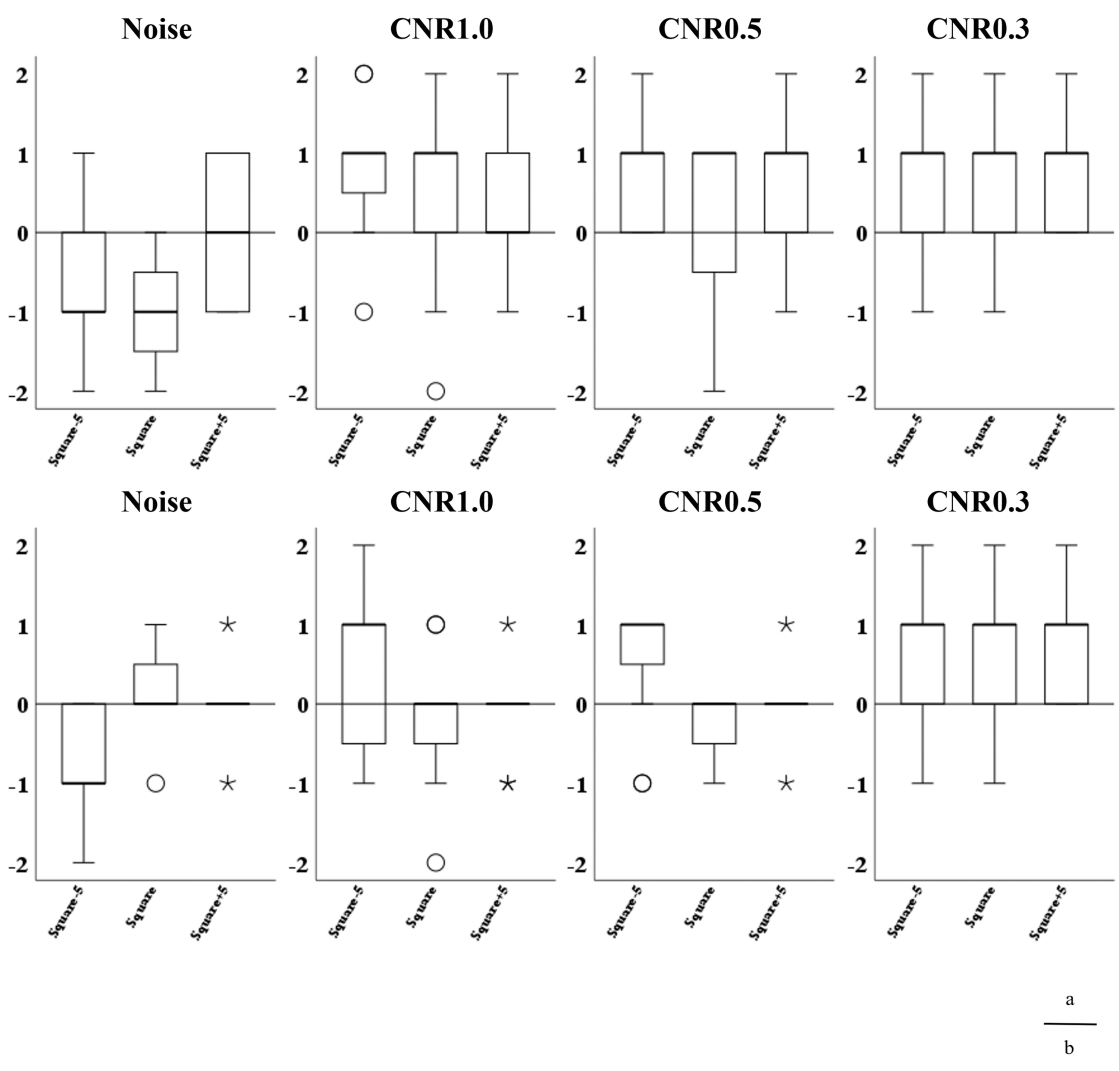
Box plots of visual evaluation scores under wide window width settings. (a) Volume scan and (b) helical scan. The box plots display the median (bold line), first and third quartiles (box), and 1.5x IQR (whiskers). Circles (o) represent outliers, and asterisks (*) represent extreme values. CNR: contrast-to-noise ratio.

**Figure 13:**
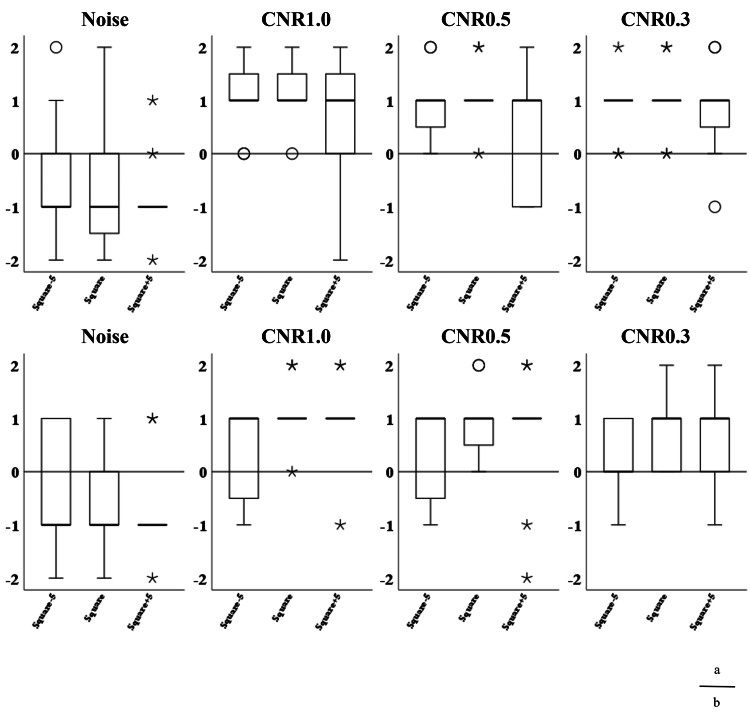
Box plots of visual evaluation scores under narrow window width settings. (a) Volume scan and (b) helical scan. The box plots display the median (bold line), first and third quartiles (box), and 1.5x IQR (whiskers). Circles (o) represent outliers, and asterisks (*) represent extreme values. CNR: contrast-to-noise ratio.

The images from the volume and helical scans are shown in Figures 8-11, and the results of the Wilcoxon signed-rank test for the wide and narrow window conditions are summarized in Table 5, respectively. The results of the visual assessment are presented in Figures 12, 13.

**Table 5:** Summary of subjective evaluation scores. Data are presented as median (first quartile to third quartile). * indicates p < 0.05 and ** indicates p < 0.01 for a significant difference from the hypothesized median of 0, based on the Wilcoxon signed-rank test.

		Wide window volume	Narrow window volume	Wide window helical	Narrow window helical
Square-5	Noise	-1 (-1 to 0)	-1 (-1 to 0)	-1* (-1 to 0)	-1 (-1 to 1)
	CNR1.0	1* (0 to 1)	1** (1 to 2)	1 (-1 to 1)	1 (-1 to 1)
	CNR0.5	1* (0 to 1)	1** (1 to 1)	1 (0 to 1)	1 (-1 to 1)
	CNR%0.3	1 (0 to 1)	1** (1 to 1)	0 (0 to 1)	0 (0 to 1)
Square	Noise	-1** (-2 to 0)	-1 (-2 to 0)	0 (0 to 1)	-1 (-1 to 0)
	CNR1.0	1 (0 to 1)	1** (1 to 2)	0 (-1 to 0)	1** (1 to 1)
	CNR0.5	1 (-1 to 1)	1** (1 to 1)	0 (-1 to 0)	1** (0 to 1)
	CNR%0.3	1 (0 to 1)	1** (1 to 1)	0 (0 to 0)	1* (0 to 1)
Square+5	Noise	0 (-1 to 1)	-1* (-1 to -1)	0 (0 to 0)	-1* (-1 to -1)
	CNR1.0	0 (0 to 1)	1 (-1 to 2)	0 (0 to 0)	1** (1 to 1)
	CNR0.5	1 (0 to 1)	1 (-1 to 1)	0 (0 to 0)	1 (1 to 1)
	CNR%0.3	1* (0 to 1)	1* (0 to 1)	0 (0 to 0)	1 (0 to 1)

Wide Window

In the noise evaluation, the median scores were zero or negative, indicating a tendency for the squared images to be rated as noisier (inferior) than the normal image. Statistical analysis revealed that the Square image under the wide window volume condition (p < 0.01) and the Square-5 image under the wide window helical condition (p < 0.05) were rated as significantly noisier than the normal image.

In the helical scan, the median scores were -1 for Square-5 and 0 for both Square and Square+5. A statistically significant difference was found only for the Square-5 image (p < 0.05), which was rated as significantly noisier than the normal image.

In the contrast evaluation for the volume scan, the median score was 1 for almost all images and contrast levels, except for Square+5 at CNR 1.0%, which had a median score of 0. Statistically significant differences (p < 0.05) were observed for Square-5 at CNR 1.0% and 0.5%, and for Square+5 at CNR 0.3%.

In the helical scan, a median score of 1 was observed only for Square-5 at CNR 1.0% and 0.5%; all other conditions had a median score of 0. No statistically significant differences were observed for any image in the helical scan.

Narrow Window

In the noise evaluation under narrow window conditions, the median score was -1 for all squared images under both volume and helical scans. Statistically significant differences (p < 0.05) were observed for the Square+5 image in both scan modes (volume and helical), indicating these were rated as significantly noisier than the normal image.

In contrast evaluation, both volume and helical scans showed more pronounced tendencies for the squared images to be rated as superior, with positive median scores across all contrast levels.

Statistically significant differences were observed in the volume scan for Square-5 and Square at CNR 1.0%, 0.5%, and 0.3% (p < 0.01), and for Square+5 at CNR 0.3% (p < 0.05).

In the helical scan, significant differences were found for Square at CNR 1.0% and 0.5% (p < 0.01), Square at CNR 0.3% (p < 0.05), and Square+5 at CNR 1.0% (p < 0.01).

## Discussion

TTF

Factors that influence the TTF include spatial resolution, reconstruction filters, and reconstruction algorithms. In this study, all squared images were generated by processing the same normal image; thus, all the influencing factors remained consistent. Therefore, the TTF was not expected to show significant changes.

In addition, the lack of impact of the squaring process on the TTF can be explained by the nature of the circular edge method used for the TTF calculation. This method involves extracting multiple radial profiles from the center of the circular phantom to obtain the edge spread function, from which the line spread function (LSF) is derived. The TTF is then calculated by applying a Fourier transform to the LSF.

Here, f(x) represents the normal image and g(x) represents the squared image.

$LSF_{\text{Normal}} = \frac{d}{dx} f(x)$

$LSF_{\text{Square}} = \frac{d}{dx} g(x)$

Here, since the squared image is defined as

$g(x) = \bigl(f(x)\bigr)^2$

$LSF_{\text{Square}} = \frac{d}{dx}\bigl(f(x)^2\bigr)$

$LSF_{\text{Square}} = 2 f(x) \cdot \frac{d}{dx} f(x)$

Thus, the LSF of the squared image takes the form of the LSF of the normal image multiplied by f(x), resulting in a change in the amplitude. However, because the TTF represents the modulation transfer characteristics across spatial frequencies, it is not dependent on absolute amplitude values. Therefore, although the amplitude of the LSF may change owing to the squaring process, it is considered that this had no effect on the shape of the TTF.

However, this result must be interpreted with caution. In this study, a replacement process (clipping) was performed to manage overflow (values > 255) and to prevent values ≤ 0 from becoming positive. The specific ROI used for this TTF measurement did not contain CT values subject to this replacement. We believe this is why no significant effect on the TTF was observed. Therefore, it cannot be concluded that the spatial resolution characteristics would remain unchanged in regions involving HU values where this clipping process does occur.

An apparent increase in the high-frequency region of the TTF was observed in the Square-5 helical scan at the 0.3% contrast level (Figure 5, bottom). However, we hypothesize that this was not a true improvement in spatial resolution, but rather a measurement artifact caused by the significant increase in noise.

Our objective noise analysis (Table 5) supports this. For this specific condition, the Square-5 process more than doubled the relative noise (CV), from 4.2% in the normal image to 9.1%. This significant increase in high-frequency noise likely contaminated the TTF calculation, as the algorithm may have misinterpreted this high-frequency noise power as a signal component.

NPS

In this study, the frequency characteristics of the NPS showed no significant differences in shape between the normal and squared images for both volume and helical scans.

The shape of the NPS depends primarily on the spatial resolution and reconstruction algorithm. Because the squared images were generated from the normal images, there were no differences in the scan conditions or reconstruction methods; thus, the frequency distribution was preserved.

By contrast, the NPS magnitude increased markedly following the squaring process.

The NPS was calculated using the following equation:

$NPS(f_x, f_y) = \frac{1}{A} \left\langle \, \left| F(u,v) \right|^2 \, \right\rangle$

$F(u,v) = \sum_{x=0}^{M-1} \sum_{y=0}^{N-1} N(x,y) e^{-j 2 \pi \left( \tfrac{ux}{M} + \tfrac{vy}{N} \right)}$

Where A is the area of the sampling region and M and N are the number of pixels in the x and y directions, respectively.

In this context, A, M, N, and $e^{-j 2 \pi \left( \tfrac{ux}{M} + \tfrac{vy}{N} \right)}$ are identical to those used in the normal image.

Therefore, the only factor influencing the NPS is the pixel value term N(x,y).

Because the squaring process nonlinearly amplifies the pixel values, it is presumed that this amplification led to an overall increase in the NPS values.

Furthermore, the slightly higher NPS observed in the helical scan may be attributed to subtle variations in the noise structure arising from differences in scanning technique [16].

CNR_LO_

The CNR_LO_ results improved for all squared images compared to the normal image, consistently ranking in the order of Square-5 > Square > Square+5 across all contrast levels in both volume and helical scans (Figure 7).

This result is determined by the ratio between the "numerator (signal difference) amplification rate" and the "denominator noise amplification rate" in the CNR_LO_ calculation (Equation 1).

Using the 1.0% nominal target level of the volume scan as an example, the numerator (signal) amplification rate increased moderately with the offset: Square-5 (approx. 102.7x), Square (approx. 112.8x), and Square+5 (approx. 122.9x).

Meanwhile, the denominator amplification rate increased sharply: Square-5 (approx. 13.7x), Square (approx. 23.4x), and Square+5 (approx. 33.2x).

Calculating the resulting CNR_LO_ improvement ratio (= Numerator Rate/Denominator Rate) yields: Square-5: 102.7x / 13.7x = approx. 7.51x improvement; Square: 112.8x / 23.4x = approx. 4.83x improvement; Square+5: 122.9x / 33.2x = approx. 3.70x improvement.

This calculation is generally consistent with the results shown in Figure 7.

Therefore, the reason Square-5 was optimal (highest CNR_LO_) is that it achieved the best trade-off: it maximized the "amplification rate ratio" (approx. 7.51x) by efficiently amplifying the signal (102.7x) while suppressing the noise amplification to its lowest level (13.7x).

In contrast, for Square+5, although the signal amplification was maximal (122.9x), the sharp increase in the denominator (noise) amplification (33.2x) far exceeded the gain in the numerator, resulting in a diminished CNR_LO_ improvement (3.70x). This suggests that the -5 HU offset represented the optimal balance point for this trade-off.

Visual assessment

In this study, although the NPS increased markedly owing to the squaring process, the visual assessment revealed only limited statistically significant differences in perceived noise. This finding suggests that the physical increase in noise was not readily perceptible, likely because of the influence of display conditions and tonal characteristics.

For instance, when WW is set to 80 on an 8-bit (256 grayscale level) monitor, one HU corresponds to approximately 3.2 grayscale levels in the normal image. In contrast, with a squared image using a WW of 4,000, one HU corresponds to only approximately 0.064 grayscale levels, making small CT value differences more difficult to perceive. This effect is particularly pronounced in low-CT regions, where noise may physically increase but appear visually suppressed.

In addition, because the squaring process amplifies higher-intensity regions more than lower-intensity regions, areas with high CT values, such as rod phantoms, exhibit larger tonal differences, contributing to improved contrast. Conversely, in low-CT regions, such as the background, amplification is minimal, which helps suppress the appearance of noise.

In the contrast evaluation, statistically significant differences were limited under the wide window condition but became evident under the narrow window condition. This was presumably because narrowing the display range decreased the HU width per grayscale level, making fine density differences easier to perceive.

However, for Square−5 in the helical scan, no significant differences were observed, even under narrow window conditions. This may be due to insufficient tonal contrast between the rod and background regions compared to the normal image. In fact, when the CT value differences between the rod and background were converted into grayscale levels, these differences corresponded to only 14, 7, and 5 levels for contrast levels of 1.0%, 0.5%, and 0.3%, respectively, which were likely too small for reliable visual discrimination.

Masuda et al. reported that noise suppression contributed to an improved low-contrast resolution [8]. In contrast, the present study suggested a different mechanism: despite the increase in NPS, perceived noise remained suppressed, whereas CT value amplification and increased tonal separation contributed to enhanced contrast resolution. In line with the findings of Nagashima et al., we also confirmed that a narrow window display improved the visibility of low-contrast regions [6].

Furthermore, applying a -5 HU offset to all pixels prior to the squaring process led to even greater contrast improvement. This indicates that combining CT value amplification with offset processing may further enhance visibility.

In this study, the evaluation was conducted under a condition in which WW was reduced by 50%. Given that the squaring process expands the dynamic range, further narrowing of the display window may improve the visibility of fine structures.

Limitations

This study had some limitations. First, only a single CT scanner model was used, and its reproducibility with other devices was not evaluated.

Second, reconstruction methods and filter kernels were not examined. Third, as this was a phantom-based study, no validation was performed using clinical images.

Fourth, the phantom's properties did not precisely reflect those of clinical reality. This study was designed with head imaging in mind (e.g., gray matter at ~40 HU and white matter at ~35 HU), but the CT values of the phantom's low-contrast rods (approx. 60 HU) and their background (approx. 50 HU) were higher than actual brain tissue. Thus, the offset values used may not be optimal for clinical applications. Furthermore, the phantom consists of homogeneous materials, unlike the heterogeneous tissue found in clinical brain images, which may have contributed to the favorable results.

Fifth, the subjective evaluation has limitations related to the reader population. The assessment was performed only by 11 radiologic technologists. While technologists are important first-line observers, the final diagnosis is made by physicians. It is uncertain whether these findings, based on visual preference, would apply to physicians or translate to improved diagnostic accuracy. Additionally, the limited number of participants (n = 11) raises the possibility that some statistically significant findings may have occurred by chance, limiting the statistical power and reproducibility of the visual evaluation results.

Sixth, the nonlinear squaring transformation poses significant technical challenges related to the data clipping required. To manage the data range, we applied a clipping process (setting values outside 0-255 HU to 0). This necessary step introduces two major limitations.

First, it impacts the interpretation of our spatial resolution (TTF) finding. Theoretically, this clipping is a non-linear operation that would be expected to alter the TTF. Our study, however, found the TTF to be unchanged. We believe this is because the specific ROI used for the TTF measurement (the edge of the CTP404 module, approx. +50 to +70 HU) fell entirely within this processing range and was thus not affected by clipping. Therefore, a key limitation is that this "unchanged TTF" result cannot be generalized to tissue boundaries involving HU values that fall outside this range (i.e., where clipping would occur).

Second, it limits the direct applicability to clinical images. While this clipping process did not cause significant artifacts in our phantom study (due to its limited HU range), clinical images contain a much wider and unpredictable range of HU values (e.g., air and bone). Applying this same clipping process to clinical images could result in the loss of important diagnostic information or the creation of new artifacts. The impact and optimization of this clipping strategy for real clinical data remain unknown.

Seventh, related to the display pipeline, mapping the extremely wide dynamic range of the squared data back to an 8-bit display for viewing is a non-trivial challenge. As our finding that the effect was diminished with a wide window suggests, the optimal display mapping (grayscale representation) to maximize the perceptual benefits of the squared transformation has not yet been established.

Eighth, the statistical analysis of our subjective evaluation involved multiple comparisons across various metrics (noise, CNR 1.0%, etc.) and conditions (volume/helical, wide/narrow). We did not apply a formal correction for multiple comparisons (e.g., the Bonferroni correction). This increases the risk that some findings identified as statistically significant (p < 0.05) may have occurred by chance, and the results of the visual evaluation should be interpreted with this in mind.

Ninth, this study focused exclusively on the squaring transformation as a method for enhancing low-contrast visibility. We did not compare its performance against other existing nonlinear contrast-enhancement mappings, such as sigmoidal functions or other advanced windowing techniques. Therefore, it is unknown whether the squaring method offers a distinct advantage over these other methods, and this comparison remains an important area for future investigation.

Further studies involving a larger number of cases and clinical images are needed to validate the findings and assess their diagnostic applicability.

## Conclusions

In this study, we investigated whether applying a nonlinear squaring process to CT images could improve low-contrast resolution using a phantom-based approach. The nonlinear squaring process improved the visibility of low-contrast regions. This subjective improvement was corroborated by an improvement in CNR_LO_. Regarding spatial resolution, although no significant changes were observed compared to the normal image in this study, the impact of clipping has not been fully elucidated. Despite a significant increase in NPS, we believe the effect of this noise was suppressed under specific display mappings.

These results suggest that the squaring process has the potential to improve the visibility of low-contrast regions in CT images. Given that the squaring process expands the dynamic range, further narrowing of the display window may improve visibility. However, the clinical utility of these findings requires validation on patient data with rigorous observer studies, including subjective evaluations by radiologists. We caution against generalizing these results as a "diagnostic aid" until such performance is validated.
